# Validity, reliability and feasibility of commercially available activity trackers in physical therapy for people with a chronic disease: a study protocol of a mixed methods research

**DOI:** 10.1186/s40814-017-0200-5

**Published:** 2017-11-23

**Authors:** Emmylou Beekman, Susy M. Braun, Darcy Ummels, Kim van Vijven, Albine Moser, Anna J. Beurskens

**Affiliations:** 1 Research Centre for Autonomy and Participation of Persons with a Chronic Illness, Zuyd University of Applied Sciences, P.O. Box 550, 6400 AN Heerlen, Netherlands; 2 Physical therapy section in multidisciplinary centre, ParaMedisch Centrum Zuid, Veestraat 28, 6134 VJ Sittard, Netherlands; 3 Department of Family Medicine, CAPHRI School for Public Health and Primary Care, Maastricht University Medical Centre, P.O. Box 616, 6200 MD Maastricht, Netherlands; 4 Research Centre for Nutrition, Lifestyle and Exercise, Zuyd University of Applied Sciences, P.O. Box 550, 6400 AN Heerlen, Netherlands; 5 Department of Health Services Research, CAPHRI School for Public Health and Primary Care, Maastricht University Medical Centre, P.O. Box 616, 6200 MD Maastricht, Netherlands

**Keywords:** Activity tracker, Pedometer, Accelerometer, Wearable, Chronic disease, Validity, Reliability, Feasibility, Physical therapy, Physical activity

## Abstract

**Background:**

For older people and people with a chronic disease, physical activity provides health benefits. Patients and healthcare professionals can use commercially available activity trackers to objectively monitor (alterations in) activity levels and patterns and to support physical activity. However, insight in the validity, reliability, and feasibility of these trackers in people with a chronic disease is needed. In this article, a study protocol is described in which the validity, reliability (part A), and feasibility from a patient and therapist’s point of view (part B) of commercially available activity trackers in daily life and health care is investigated.

**Methods:**

In part A, a quantitative cross-sectional study, an activity protocol that simulates everyday life activities will be used to determine the validity and reliability of nine commercially available activity trackers. Video recordings will act as the gold standard. In part B, a qualitative participatory action research study will be performed to gain insight in the use of activity trackers in peoples’ daily life and therapy settings. Objective feasibility of the activity trackers will be measured with questionnaires, and subjective feasibility (experiences) will be explored in a community of practice. Physical therapists (*n* = 8) will regularly meet during 6 months to learn from each other regarding the actual use of activity trackers in therapy. Therapists and patients (*n* = 48) will decide together which tracker will be used in therapy and for which purpose (e.g., monitoring, goal setting). Data from the therapist’ and patients’ experiences will be collected by interviews (individual and focus groups) and analyzed by a directed content analysis. At the time of submission, selection of activity trackers, development of the activity protocol, and the ethical approval process are finished. Data collection and data processing are ongoing.

**Discussion:**

The relevance of the study as well as the advantages and disadvantages of several aspects of the chosen design are discussed. The results acquired from both study parts can be used to create decision aids that may assist therapists and people with a chronic disease in choosing a suitable activity tracker, and to facilitate use of these activity trackers in health care settings.

**Trial registration:**

Ethical approval has been obtained from two medical-ethical committees (nr. 15-N-109, 15-N-48 and MEC-15-07).

## Background

In Western countries there is an increase in incidence and prevalence of chronic diseases. The most prominent chronic diseases are chronic obstructive pulmonary disease (COPD), cardiovascular diseases, type 2 diabetes and oncological diseases [1, 2]. Regular physical activity is recommended in several evidence-based guidelines because of its positive influence on the development of the chronic disease and lower risk of comorbidity and premature death [3–6]. However, many of the patients with a chronic condition are inactive. For healthy people, an average daily physical activity of 30 min at a moderate intensity is suggested and the World Health Organization recommends similar amounts for people with chronic diseases [7–9]. It is also advised that when patients cannot meet the activity levels suggested in guidelines for healthy people, “*they should be as physically active as their abilities and conditions allow*” ([10] p. 30).

For the development of an individualized activity plan, information on a patient’s physical activity level is required. This includes also information on physical activity during activities of daily living, such as vacuum cleaning and carrying groceries, as these activities involve a physical activity component as well [11]. Questionnaires, diaries, and daily activity logs assessing physical activity have limited reliability and validity, tend to overestimate most activities while underestimating low-intensity activities, and are time-consuming to fill in [12, 13]. For patients and healthcare professionals, more objective and feasible measurement tools are needed.

A potential alternative could be activity trackers, which allow people to gain insight in their own physical activity behavior [14]. The use of activity trackers for self-monitoring is gaining popularity in consumers [15]. In the Netherlands, 23% of the population occasionally track their physical activity and approximately 5% do this continuously [16]. There are many different commercially available activity trackers on the market, with a wide variety in costs, ease of use and underlying algorithms to determine physical activity parameters [17, 18]. For example, costs range from freely available applications for the smartphone to more expensive devices that contain multiple sensors. There is also a variety in which activities trackers measure. Most trackers are capable of measuring the number of steps, whereas for example the measurement of cycling-movements is rare.

The body of literature on clinimetric quality of commercially available activity trackers in healthy participants is growing [19–21]. For example, a recent study of Case et al. showed that a selection of commercially available activity trackers measured the number of steps on a treadmill quite accurately in healthy participants [22]. However, little is known about which (types of) activity trackers provide valid and reliable results in everyday life situations in people with chronic diseases [17, 23].

In addition, the actual use of the trackers in health care needs attention. Studies have been published regarding the feasibility of general health programs which include an activity tracker [24]. However, little is known about the feasibility of activity trackers from the patients’ and physical therapists’ point of view and how integration in health care could be facilitated. The same activity tracker can be assessed less or more feasible depending on the characteristics of the users and the aim for which the tracker is used [24, 25].

The first aim of this study is therefore to investigate if commercially available activity trackers are valid and reliable for people with a chronic disease (part A). The second aim is to identify which factors from a patient and therapist’s point of view influence the feasibility of activity trackers in health care (part B). In this article, a study protocol is described in which the validity, reliability, and feasibility of commercially available activity trackers is investigated.

## Methods

This research project consists of two parts (A and B). In part A, a quantitative cross-sectional study will be used to determine the validity and reliability in an activity protocol that simulates everyday life activities. In part B, a qualitative participatory action research study will be performed to gain insight in the feasibility of activity trackers in health care. At the time of submission, selection of activity trackers, development of the activity protocol and the ethical approval process are finished. Data collection and data processing are ongoing. Ethical approval has been obtained from the local medical-ethical committee (nr. 15-N-109, 15-N-48 and MEC-15-07).

### Selection of activity trackers

In collaboration with therapists the following selection criteria were used to select the activity trackers included in this study. The activity tracker should:

1. Cost less than 150 euros.
2. Have no monthly costs for a subscription.
3. Have real-time feedback to the user.
4. Have no chest strap for heart rate measurements.

After an extensive online search for all commercially available activity trackers that were on the market by May 2015, a total of 72 trackers were found eligible (a data file of the selection can be found on a Dutch website: http://www.meetinstrumentenzorg.nl/Home/Sources, under the purple button). From these trackers a maximum of ten trackers was picked for the study. To ensure that the scope of different system requirements was covered, trackers were randomly selected in a second round based on following criteria:

5. Variety of wearing places (e.g., belt, wrist),
6. Types of activity trackers (i.e., pedometers, accelerometers and smartphone applications)
7. Variety of tracked activities (e.g., walking, cycling).

Hence, nine activity trackers were selected and will be investigated in this research project, covering the variety in wearing place, type, and activities measured (Table 1).

**Table 1:** Overview of the selected activity trackers with specific characteristics regarding manufacturer, type of tracker, wearing position, and outcome variables

Activity tracker	Manufacturer	Type	Wearing position	Outcome variables
Accupedo Step Counter	Corusen LLC	App	Belt	A
Activ8	Remedy Ltd	Accelerometer	Trouser pocket	A, B, C
Digi-Walker CW-700	Yamax Coorporation	Pedometer	Belt	A, C
Flex	Fitbit Inc.	Accelerometer	Wrist	A, C
Lumo Back	Lumo BodyTech, Inc.	Accelerometer	Lower back	A, C, D
Moves	ProtoGeo	App	Trouser pocket	A, C
One	Fitbit Inc.	Accelerometer	Belt	A, C
UP24	Jawbone	Accelerometer	Wrist	A, C
Walking Style X	Omron Healthcare Europe B.V.	Accelerometer	Belt	A, C

*A* number of steps, *B* time spent lying, sitting, standing, walking, running, and cycling, *C* active minutes, *D* number of sit to stand transitions

#### Part A: validity and reliability of selected activity trackers

In a cross-sectional study, the activity tracker measurements will be compared to video recordings of activity performance to determine the validity of the trackers. Repeated measurements will be used to determine the test-retest reliability of the activity trackers.

### Participants

**Fig. 1:**
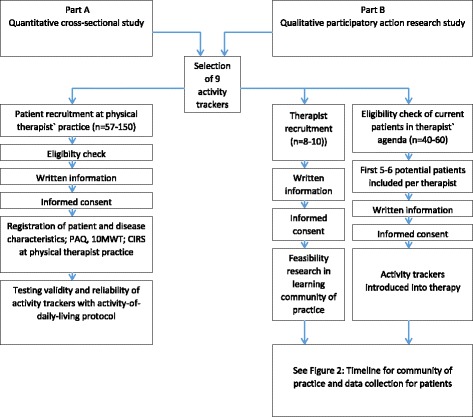
Flow chart of the study (part A and part B)

People with one or more of the following chronic diseases will be included at three physical therapy practices in the south of the Netherlands: cancer, cardiovascular disease, COPD, diabetes mellitus, arthritis, or chronic pain. All conditions should be diagnosed by a physician. The existence of this diagnosis will be verified by the physical therapists. For cancer, all types of malignity and both curative as well as palliative treatment phases are considered; for cardiovascular disease, no type or severity is defined for inclusion; for COPD, all Global Initiative for Chronic Obstructive Lung Disease (GOLD) stages are considered; for diabetes, type 1 or 2 are considered; for arthritis, all body locations are considered; and in the case of chronic pain, the pain should be lasting more than 3 months at the time of inclusion. No age restrictions apply. To be included in the study, participants have to have sufficient understanding of the Dutch language and they have to be able to perform light to moderate physical activity. Potential participants that use a walking aid or have an asymmetric walking pattern will be excluded from participation. All participants will be given written information about the study, will have the opportunity to ask questions, and will be able to consider participation for at least 1 week. All participants are asked to provide written informed consent. A flowchart of the study is presented in Fig. 1.

### Power calculation

A minimum sample size of at least 50 respondents is generally recommended for the assessment of test-retest reproducibility [26]. To determine the required number of participants, power calculations for validity and reliability were performed. For type one error of 0.05 and power 0.90, a conservative sample of 25 participants wearing an activity tracker type would be needed to detect differences in the number of steps between trackers (based on a SEM of 1.9 or to identify a correlation of 0.5 as being statistically significant different from no correlation, assuming a one-sided type one error) [27].

Each participant will wear three to four different activity trackers. Therefore, 113–150 participants for reliability and a minimum of 57–75 participants for validity with an equal spread among the six chronic subpopulations are considered to be sufficient.

### Activity protocol

The developed activity of daily living protocol is based on principles of other activity protocols used in earlier performed validation studies of activity trackers [28–31], but it includes only activities that are representative for everyday activities. The protocol is presented in Table 2. To avoid unnecessary tiring of the participants, two versions of the protocol were developed. Both protocols include activities like vacuum cleaning while walking, sitting, and carrying a shopping bag. The short version of the protocol does not include lying on a bed, vacuum cleaning on the spot, and three additional periods of standing, shortening the execution time with 9 min.

**Table 2:** Overview of the long and short activity protocol.

Station	Activity type	Duration activity, repetitions or walking distance	Included in short version
1	Standing	1 min	X
	Simulated cleaning of windows	1 min	X
	Walking weaving around cones	7 m	X
2	Sitting on a chair	2 min	X
	Standing	1 min	
	Vacuum cleaning on the spot	1 min	
	Vacuum cleaning while walking	1 min	X
	Walking weaving around cones	7 m	X
3	Walking up and down stairs (3 or 4 steps)	3 times	X
	Lifting a 1 kg object and placing this at a table	1 min	X
	Walking in a straight line	7 m	X
4	Lying on a bed	6 min	
	Sitting on a chair	5–10 min	X
	Standing	1 min	
	Walking in a straight line while carrying a shopping bag (content: 2.5 kg)	2 times 7 m	X
5	Walking sideways along a 2 m kitchen counter	3 times 2 ways	X
	Standing	30 s	
	Walking in a straight line	7 m	X
6	Cycling (50–60 rpm at 30 watt)	3 min	X
Total time		28–33 min	19–24 min

The long version of the protocol included all mentioned activities. The third column shows which activities are included in the short version of the protocol

### Data collection and procedure

Before the start of the activity protocol, general participant characteristics (i.e., gender, age and diagnosed chronic disease) will be registered. The Physical Activity Questionnaire [32, 33] will be used to measure the daily physical activity level of participants. The mean of three 10-m walk tests (10MWT) is used to measure the participant’s comfortable walking speed. During the test, therapists will observe whether participants have a symmetrical gait pattern. The Cumulative Illness Rating Scale (CIRS) [34] will be filled out to gain a cumulative score on comorbidity count and severity. Additional disease specific characteristics will be registered. For instance, for COPD, the Global Initiative for Chronic Obstructive Lung Disease (GOLD) stage will be noted, for cancer, the type of malignity, presence of metastases, type of treatment, number of weeks post-treatment, and treatment phase (i.e., curative or palliative) will be registered. All abovementioned information will be collected by the physical therapist in the practice on the day of testing.

Each participant will wear three different activity trackers during the performance of the activity protocol, executed at the physical therapy practices. Treating therapists will decide whether the participant performs the long or short protocol, which will be performed at a comfortable speed for the participant. Breaks are possible if participants require them. The performance of the activity protocol will be recorded with a video camera, which is considered to be the gold standard [35]. Participants will perform on the activity protocol twice, interspersed with a 15–30-min rest period. In case a participant is unable to perform the protocol twice on the same day, the second measurement will be done the day after the first measurement.

### Data analysis

The statistical software Statistical Package for the Social Sciences (SPSS) (version 23) is used for the analyses. Data of all subjects are checked for missing values, distribution (with the Kolmogorov-Smirnov test of normality), and outliers.

Each video will be analyzed by at least one researcher from an instructed pool of seven assessors. The seven assessors will use a standardized written assessment protocol, and all assessors will be trained by the same senior researcher. The two first video recording assessments will be checked by the senior researcher to ensure standardization of the measurement method. In addition, a randomly chosen sample of recordings (10%) will be analyzed by a second assessor, to allow for reliability analysis of the gold standard measurement method. The assessors will register the time period of the activities and the number of steps taken by the participant. For the number of steps taken, two different ways of counting will be used. First, a step will be counted as one when the entire foot is lifted from the floor. As we expect some participants to shuffle or pivot on the spot, steps will also be counted when participants replace their foot. This might be a forward, backward, sideways, or upwards move, with or without constant floor contact. No steps are counted when only part of the foot is lifted and put down on the floor at the same location, or when a weight shift happens while the position of the foot stays on the same spot.

To determine the validity, a Pearson or Spearman correlation coefficient (depending on data distribution) will be calculated between the outcome measures of the activity tracker and the outcomes of the video analysis. In case the first performance contains missing values after video analysis, the second performance of the protocol will be used to assess the validity.

An outcome measurement of an activity tracker is considered valid in this study population if *r* ≥ 0.7 [27]. The validity results will be visually displayed in graphs.

To determine the reliability of the activity trackers, Intraclass Correlation Coefficients (ICC agreement), Standard Errors of Measurement (SEM agreement) and lower limits of agreement from Bland-Altman plots will be calculated between the first performance and second performance on the activity protocol within each participant, for each tracker. All ICCs will be calculated with a single measures random approach. This is considered to be the most conservative option, since the position of the tracker may slightly deviate during the second recordings of the activity protocol**.**

#### Part B: objective and subjective feasibility of activity tracker use in physical therapy

In a qualitative participatory action research study, the feasibility of the nine activity trackers from both a therapist and patient’s perspective will be investigated in which we make a distinction between objective and subjective feasibility [36]. Objective feasibility is defined as the (more) objective characteristics of the activity trackers: costs of purchase and use, availability, complexity and accessibility of the trackers, required skills (e.g., training), required time for use and interpretation of the data and technical performance (e.g., errors, battery duration, waterproofness and compatibility). Subjective feasibility is defined as the experiences of healthcare professionals and patients with the daily use of activity trackers (e.g., attitude about the advantages and disadvantages and understanding of the tracker) and the usefulness of the activity tracker in the healthcare process (e.g., motivation for use, therapy-related goals).

### Therapists: selection and procedure

Via purposive sampling, eight to ten physical therapists will be selected with a variety in age, gender, education, years of work experience, and additional education from three physical therapy practices in the community in the south of the Netherlands (Fig. 1).

**Fig. 2:**
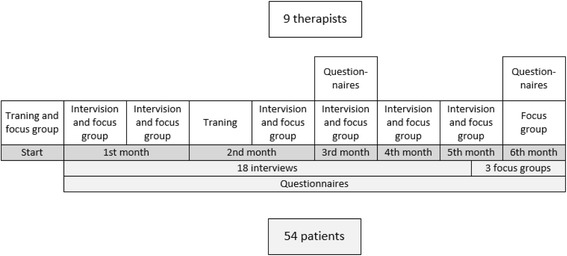
Timeline for community of practice for therapists and data collection for participating patients

During a time period of 6 months, the participating therapists will form a learning community of practice [37]. This community of practice is at the heart of the participatory action design because it involves the active involvement of the intended users. The aim of this community is developing and exchanging knowledge about the use of activity trackers in therapy through the experiences of the members. This will be done through a series of eight interactive training sessions, feedback sessions, and focus group interviews (see Fig. 2 for the timeline). Because it is not feasible for the participating therapist to acquire full knowledge and skills needed for the use of all nine trackers in 6 months’ time, each therapist will get a set of four different activity trackers and each tracker will be used by at least four therapists. In this way each therapist gets a diverse selection of trackers (e.g., wearing places, type of tracker).

The first training session gives therapists basic knowledge about activity trackers in general and the characteristics of the nine selected activity trackers. Examples are wearing places, additional required devices and measurement options. After the first session, therapists will be asked to apply the activity trackers in therapy for patients with a chronic disease. There will be no protocol about how to use the activity trackers; therapists are free to use any of the four activity trackers in the way they want to use it in daily care. This will create a real-life situation of the implementation of activity trackers in therapy.

In the first 2 months of the time period in part B, the therapists will get together for three feedback sessions and one training session. The topics of the feedback sessions will depend on the experiences of the therapists. The training session will take place in the second month, in which the participants will receive information about behavior influencing theories applied to the use of activity trackers in healthcare. In the remaining 4 months, the therapists will get together once a month. The content depends again on the experiences gained by the therapists. Each session will last 90–120 min.

### Data collection and analyses

Data will be collected during the eight group activities. Each session will be audio recorded, and field notes will be taken on the main topics discussed and on the nonverbal responses of the participants. All audio recordings from interviews will be transcribed verbatim by an independent researcher. The data will be analyzed using directed content analysis. A literature search on feasibility studies about activity trackers in healthcare has resulted in sensitizing concepts around objective and subjective feasibility from therapists and patients’ perspectives. If parts of the transcripts appear not to suit the existing (sub)categories, a new (sub)category will be added [38]. Two interviewers will code the data. The first two interviews will be coded by both researches independently, first to test the coding framework and adopt it if necessary and second to fine-tune coding between the two researchers. Differences in coding and interpretation will be discussed to find the “best suitable” interpretations. Then, the researchers will code two transcripts individually and meet for a coding session, also to refine the coding scheme where necessary. Continuous fine-tuning will continue until all interviews are coded. Anonymity and confidentiality will be secured by anonymously transcribing and analyzing the audio recordings.

After 3 and 6 months, three questionnaires will be used to gather information about each of the four activity trackers the therapist is using. A self-developed questionnaire will be used to gather information about objective feasibility aspects such as technical performance of the activity trackers. A modified version of the Post Study System Usability Questionnaire (PSSUQ; [39]) will be used to assess how participants evaluate the activity tracker and the related computer dashboard or smartphone app. The PSSUQ is modified since some of the original questions of Lewis are applicable for activity trackers or the therapy setting (original items 3, 9, 10, and 17 are deleted from the questionnaire [39]). Also, the word “system” is replaced with “activity tracker”. The User Experience Questionnaire (UEQ) [40] will be used to gain insight into the experiences the participants have while using the activity trackers, related to the domains “pragmatic”, “hedonic identification”, “quality-stimulation”, as well as “beauty” and “goodness”.

### Patients: selection and procedure

Via purposive sampling 40–60 patients with a variety in age, gender, technical skills (e.g., familiarity with self-monitoring, using the computer) and chronic disease will be selected. The in- and exclusion criteria are the same as in part A. In addition, the highest level of education of a participant is registered as an indicator of the socio-economic status of the participant. Physical therapists have direct access to the target population for this study (in the Netherlands physician referral is not necessary). Physical therapists check the official diagnosed diseases as well as comorbidity with the physician; this is standard procedure for physical therapist in the Netherlands. With the start of the study, physical therapists will check whether their current patients fit the study inclusion criteria. Eligible patients will be approached and asked by their participating therapists in the order of appearance in the consultation agenda. Each therapist will select five to six patients with a chronic disease to participate in the study.

Potential participants will be given written information about the study and will be able to consider participation for a least a week. All participants are asked to provide written informed consent 1 week after they received written information and before the activity tracker is introduced into the therapy. A flowchart of the study is presented in Fig. 1. The therapist and patient decide which activity tracker out of the four available trackers will be used, for which duration and for which purpose.

### Data collection and analyses

Individual and focus group interviews will be held with participating patients at the patients home or in the physical therapy practice (whatever was more convenient for the patient). During individual interviews, based on a semi-structured interview guide, participants will be invited to share their experiences with the activity tracker within the therapy process and in daily living. Each interview will last for about 30 min and a memo recorder will be used to record the interview. The interviewer will also take field notes, describing the main topics of the interview and any notable events during the interview. The aim is to interview 15–20 participants. Based on the content of the individual interviews, the questioning route for the focus group interviews will be developed. Each focus group interview will last for about 90 min and will be recorded with memo recorders. Field notes will be made on the main topics and the nonverbal responses of participants. The aim is to include a total of 18–24 participants divided over three focus group interviews. Participants will be included until data saturation is found. To establish data saturations, agreement sessions will be planned regularly within the research team. The qualitative data will be analyzed in the same way as the data of the therapists.

When the period of use of the activity tracker is finished, patients will be asked to fill out the PSSUQ and the UEQ. Questionnaires are distributed among the physical therapists to have them filled out by the patients at their home or at the practice. The PSSUQ is modified slightly different in comparison to the version filled out by the therapist (item 4, 5, 8, 9, 10, and 14 of the original questionnaire are removed [39]). For all questionnaires, descriptive statistics will be used for each activity tracker. As participating patients fill out all questionnaires twice, differences over time will be investigated.

## Discussion

In this article a study protocol is presented to investigate the validity, reliability, and feasibility of commercially available activity trackers in people with a chronic disease. The results of part A will give an indication of which of the included activity trackers measure the investigated parameters of physical activity valid and reliable. It is expected that the results of part B will provide insight in factors from a patient and therapist’s point of view influence the feasibility and use of activity trackers in daily life and in physical therapy.

### Discussion points with regard to the design of the study

During the design of this study, the research group discussed several aspects of the research protocol. Discussion points included 1. The selection of the activity trackers; 2. The choice for target populations; 3. Developing the protocol with daily activities; 4. Defining a step and 5. Gaining insight into use of trackers in daily care.

#### Selection of the activity trackers

The selection criteria for the activity trackers were based mainly on accessibility (in costs) and ease of use for vulnerable populations in health care. Both patients and health care professionals should be able to gather information on levels of activity quickly (e.g., no necessary use of software or other product to interpret the meaning of the data). Although the final choice for the selected trackers in the second round was to some extent random, we did make sure that we had a varied selection that is representative for the majority of trackers available on the market that time. We for instance included pedometers, accelerometers, and Apps and varied locations of where to wear the trackers [41]. We also checked whether the activity trackers are already frequently used and how they are being rated by consumers (e.g., on websites). This representation of activity trackers is of importance, because new activity trackers are developed rapidly and are launched more frequently than ever before. For research, it is difficult to keep up with these developments. Therefore, this study does not only aim to look at the specific features of the included devices, but also to gather information about more general implementation features which will still be applicable to wearables in healthcare over a longer time [42].

#### Choosing the target populations

Algorithms to detect for instance steps and active minutes in activity trackers are designed for symmetrical walking gait. Therefore, the use of trackers in participants with asymmetrical walking gait, might introduce bias in determining the validity and reliability of activity trackers. Even though asymmetrical walking gaits are common in for example neurological diseases, we decided to exclude this condition [43]. The same accounts for walking with a one-sided walking aid (e.g., cane).

#### Content and structure of the daily activity protocol

In order to assess the validity and reliability of the commercially available activity trackers in everyday life situations a standard protocol needs to be used with in it only (simulated) daily activities. However such a protocol is lacking [35], but we do have information on how validation protocols are structured in general [28, 29, 44]. Basic elements of a validation protocol are among others, a “description of the socio-demographic characteristics of the target population”, “a measuring protocol”, and “a gold standard” (external criterion). Important criteria for the “measuring protocol” reported in the literature are the length of the protocol (about 30 min) and a standardized course in which free-living conditions are performed or simulated. Based on this information, the researchers developed a measuring protocol, which will be used for all trackers to both enable comparison between the trackers and assess their validity and reliability in daily activities. The added value of the developed protocol for future other studies, has to be established. A validation study by Rabinovich et al. suggested that activity trackers should be used to assess the activities (i.e., movements) of patients in terms of amount and/or intensity of activity and that greater weight should be given to direct monitor outputs (e.g., steps). In this light standardized protocols to validate the number of steps in activity of daily living protocols are needed [45]. To avoid unnecessary tiring of patient with a bad condition, we developed also a short version of the protocol that contained most of the activities.

#### Determining when to count a step

To determine the validity of the tracker, the research group described a step as follows: We consider a *person to make a step when the entire foot is cleared of the floor and is placed back on the floor again* **or** *when the participant replaces the entire foot without foot clearance during shuffling*. The first part of the description is the more technical definition of walking. We assume this part is used in most validation studies (e.g., Storm and colleagues (2015): steps based on heel strike and toe-off [46]). However, in people with a chronic disease, shuffling gait is seen frequently [47]. As moving with a shuffling gait adds to the daily physical activity of a person, an activity tracker should be able to record this activity if the user wants to gain a complete picture of walking activities. Therefore, the information on which activity trackers are able to count steps accurately according to the second part of the description is important for use in therapy. Using both definitions of steps, validity of the activity trackers can be established when shuffling gait is and is not included.

#### Gaining insight into use of trackers in daily care through participatory community of practice

Several studies have been performed in which the feasibility of activity trackers has been assessed including their effect on the activity behavior of the users [48, 49]. Results show that activity trackers have a (short term) positive effect on becoming more active. Part of the effectiveness is explained by an increase in self-management. Motivation, opinions, and intentions of the users (e.g., patients) are of great importance to successfully increase self-management [50]. In addition, the support of a professional (e.g., therapist) may facilitate the self-efficacy of the client [51].

Less is known about the feasibility of activity trackers in daily care [42]; there is a need for studies that evaluate factors from patients and therapist’s point of view [24]. We make a distinction between the more objective features of the devices (e.g., battery duration) and the subjective feasibility such as the experiences of patients and therapists (advantages and disadvantages) and usefulness in the healthcare process. This requires a qualitative in depth approach and the intensive involvement of both therapists and patients [42]. An action research approach (e.g., participatory community of practice) is warranted, because we especially want to know how activity trackers can be implemented in daily care, for instance by embedding the potential use of the trackers in the clinical reasoning process. Although the choice for the design ensures the research to be as closely linked to the real-life situation as possible, it also inhales the risks of any open self-guiding approach. Within the community of practice the process and content of the eight meetings will mainly be guided by the input (e.g., experiences, opinions, beliefs) of the participating therapists. Although researchers can influence the process a little, it is not given that at the end all factors for successful use of the trackers within health care will all be identified.

### Conclusion

Information about commercially available activity trackers is widely available. However, no information is available about their quality and use in healthcare settings. Therefore, the combined information from both study parts can be used to create decision aids that may assist patients and therapists in choosing which tracker for which patient, in what treatment stage and for what purpose.

## Abbreviations

10MWT: 10-m walk test
CIRS: Cumulative Illness Rating Scale
COPD: Chronic obstructive pulmonary disease
GOLD: Global Initiative for Chronic Obstructive Lung Disease stage
ICC: Intraclass correlation coefficients
PSSUQ: Post Study System Usability Questionnaire
SEM: Standard errors of measurement
SPSS: Statistical Package for the social sciences
UEQ: User Experience Questionnaire
